# Innovative Oral Treatments of Uterine Leiomyoma

**DOI:** 10.1155/2012/943635

**Published:** 2012-02-16

**Authors:** Mohamed Sabry, Ayman Al-Hendy

**Affiliations:** ^1^Center for Women Health Research (CWHR), Meharry Medical College, Nashville, TN 37208, USA; ^2^OB/GYN Department, Faculty of Medicine, Sohag University, Sohag 82524, Egypt; ^3^Center for Women Health Research, Department of Obstetrics and Gynecology, Meharry Medical College, George Hubbard Hospital, 1005 Dr. D.B. Todd Jr. Blvd, 5th Floor, Room 5131C, Nashville, TN 37208, USA

## Abstract

Uterine fibroids (leiomyoma), the benign tumors of the uterine wall, are very common cause of morbidity in reproductive age women usually in the form of excessive vaginal bleeding, chronic pelvic pain, miscarriage and infertility. These tumors are the leading indication for hysterectomy in the United States. Uterine fibroids are about 4 times higher in blacks compared to whites and constitute a major health disparity challenge. The estimated cost of uterine fibroids is up to $34.4 billion annually. Additionally, women who suffer from this disease and desire to maintain their future fertility have very limited treatment choices. Currently, there is no effective long-term medicinal treatment for uterine fibroids. While surgery has traditionally been the gold standard for the treatment of uterine fibroids, there is growing interest towards orally administered medications for the management of leiomyoma-related symptoms. In this paper, we will discuss these promising innovative oral medical treatments in detail.

## 1. Introduction

Uterine leiomyomas are the most common benign pelvic tumors in women [1, 2]. They are monoclonal tumors of the smooth muscle cells of the myometrium and consist of large amounts of extracellular matrix that contain collagen, fibronectin and, proteoglycan [2, 3]. A thin pseudocapsule that is composed of areolar tissue and compressed muscle fibers usually surrounds the tumors [4]. Leiomyomas may enlarge to cause significant distortion of the uterine surface or cavity. Dark skinned women, such as African Americans, also had higher numbers of leiomyomas and tended to have larger uteri, which in turn may explain the higher incidence of in-hospital complications or blood transfusion requirements in AA women compared to white women [5, 6]. The overall incidence of uterine leiomyomas is estimated to be 3-4 times higher in African American women compared to Caucasian women [7–10]. Recent data have also confirmed that the age-standardized rates of ultrasound- or hysterectomy-confirmed leiomyoma were significantly higher in black women compared to white women [11]. Although they are benign, they commonly result in severe symptoms, such as heavy, irregular, and prolonged menstrual bleeding as well as anemia. Uterine leiomyomas have also been associated with numerous other medical disorders, such as infertility, recurrent abortion, and preterm labor [12]. These clinical complications negatively impact women's health. Uterine leiomyomas are the most cited indication for the more than 600,000 hysterectomies that are performed in the US annually, and this major surgery is associated with morbidity and mortality as well as a huge economic impact on healthcare delivery systems that is estimated to be approximately $34.4 billion/year [13] (Table 2).

## 2. Current Treatment Options for Uterine Leiomyomas

Treatment options for leiomyoma vary; treatment strategies are typically individualized based on the severity of the symptoms, the size and location of the leiomyoma lesions, the patient's age and their chronological proximity to menopause, and the patient's desire for future fertility. The usual goal of therapy is the relief of the symptoms (Table 1). The treatment options range from the use of acupuncture (ancient Chinese method) to the total removal of the uterus and its myoma contents (hysterectomy) [14]. To date, there is no definitive oral therapeutic agent for the treatment of uterine leiomyomas, which is a reflection of the remarkable lack of randomized clinical trial data that demonstrate the effectiveness and safety of medical therapies in the management of symptomatic leiomyomas [15].

## 3. Oral Medical Agents for the Treatment of Uterine Leiomyomas

Currently, there are no definitive FDA-approved agents for the oral medical treatment of uterine fibroids. However, there are several candidate agents that can be used in addition to other approaches in the management of this common benign tumor.

However, there are several candidate agents that can be used with varying degrees of success. Increasing knowledge of the mechanism of action of more recent candidate agents such as Vitamin D, Green tea extract, and Elagolix (oral GnRH antagonist) as well as that of older agents such as selective estrogen receptor modulators (SERMs), antiprogestins, aromatase inhibitors, cabergoline, danazol, and gestrinone may lead to the development of an oral agent with the ability to shrink leiomyoma size with minimal side effects.

This consequently will be discussed.

### 3.1. Vitamin D (VitD)

Data from our laboratory demonstrate that Vitamin D (VitD) is an antifibrotic factor and inhibits growth and induces apoptosis in cultured human leiomyoma cells through the downregulation of PCNA, CDK1, and BCL-2 and suppresses COMT expression and activity in human leiomyoma cells [16–18]. We have also recently demonstrated similar effects in the Eker rat model of uterine fibroids [19]. Another group in Finland demonstrated that Vitamin D inhibits growth of both myometrial and leiomyoma cells in vitro [20]. The growth inhibition was concentration dependent and the level of inhibition was statistically significant with the concentration of 1000 nM.

In a separate study from our group, the correlation between low serum levels of VitD and the increased risk of having symptomatic uterine fibroids were evaluated [21, 22]. We measured both the biologically active 1, 25 dihydroxyvitamin D3 and the precursor 25-hydroxyvitamin D3 in the serum from African American and white women with fibroids as well as normal healthy controls. Interestingly, then observed that 1, 25 dihydroxyvitamin D3 is significantly lower in women with fibroids compared to normal healthy controls; additionally, there have been detected lower levels of total serum 25-hydroxyvitamin D3 in women with fibroids compared to healthy controls. These findings were observed both in African American women and in Caucasian women.

**Figure 1:**
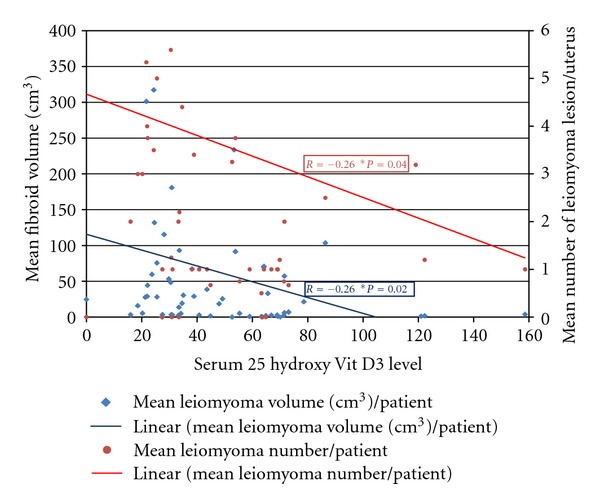
Serum Vitamin D3 level (nmol/L) inversely correlates with both mean volume and number of uterine fibroids.

The aim of the study was to determine whether serum levels of VitD correlated with disease severity in women with symptomatic uterine fibroids. The study population consisted of 67 patients who had detailed repeated pelvic ultrasound evaluations over a 2-year period with specific measurements of the total uterine volume and the volume of the individual leiomyoma lesions. The patients also had detailed laboratory analysis including serum 25 hydroxy Vit D3 levels. As shown in (Figure 1), a statistically significant negative correlation between the low serum VitD levels and the total uterine leiomyoma volume (*P* < .05) as well as the number of leiomyoma lesions/uterus (*P* < .05) was detected [23]. Taken together, our preliminary results suggest a strong dose-response correlation between lower serum VitD levels and increased severity of uterine fibroids. This presents an opportunity for the potential use of VitD or its potent analogues as novel treatment options or for the prevention of uterine fibroids. 

To date no randomized controlled trials had been implemented to prospectively assess the efficacy of VitD in the management of uterine fibroids.

## 4. Epigallocatechin Gallate (EGCG): Green Tea Extract

Tea is one of the most widely consumed beverages all over the world. Both the green tea and the black tea are derived from the leaves of the plant “Camellia sinensis”, the most significant components of which are phytochemicals, of which Green tea is thoroughly studied for its health benefits. 

A typical green tea beverage, prepared in a proportion of 1 g leaf to 100 mL water in a 3-min, brew, usually contains 250–350 mg tea solids, and catechins account for 30–42% of the dry weight of the solids [24]. It has been demonstrated that tea constituents exhibit various biological and pharmacological properties such as anticarcinogenic, antioxidative, antiallergic, antivirus, antihypertensive, antiatherosclerosis, anticardiovascular disease and antihypercholesterolemic activities [25, 26].

The major green tea catechins are epigallocatechin-3-gallate (EGCG), epigallocatechin (EGC), epicatechin-3-gallate (ECG), and epicatechin. Catechins are a group of bioflavonoids that exhibit antioxidant and anti-inflammatory capacity. Chemically, catechins are polyhydroxylated with water-soluble characteristics [27]. Epigallocatechin gallate (EGCG), which is the principal catechin, comprises >40% of the total polyphenolic mixture of green tea catechins [28]. Grapes also contain polyphenols and catechins such as EGCG [29]. Epigallocatechin gallate exhibits various biological activities including potent antioxidant and anti-inflammation capacity [30].

EGCG appears to block each stage of tumorgenesis by modulating signaling pathways involved in cell proliferation, transformation, inflammation, and oxidative stress, which are clearly involved in pathogenesis of various tumors including uterine fibroids [31]. In our laboratory, the effect and potential mechanisms of EGCG action on human leiomyoma (HuLM) cells [32] were studied: cell proliferation and apoptosis were assayed; the protein levels of PCNA, CDK4, BCL2, and BAX were examined by Western blot analysis, and it was found that EGCG inhibits the proliferation of HuLM cells and induces apoptosis. These results suggest that EGCG may be a potential anti-uterine fibroid agent acting through multiple signal transduction pathways [33]. 

 Additional validation of these findings was achieved using orally administered EGCG to shrink preexisting subcutaneous leiomyoma lesions in immune-compromised mice [32]. Previous studies have shown that EGCG inhibited the growth of various human cancer cells, such as epidermoid carcinoma cells [34], hepatoma cells [35], prostate carcinoma cells [36], and breast cancer cells [37]. Those findings motivated us to initiate a currently ongoing double-blind placebo-controlled clinical trial (phase II trial) to evaluate the promising clinical role of EGCG in women with symptomatic uterine fibroids.

## 5. GnRH Antagonists

The 3rd-generation GnRH antagonists display a more tolerable side effect profile compared to the first-generation GnRH antagonists (histamine release and severe allergic reactions) and the second generation GnRH antagonists (allergy and gel formation); some of the GnRH antagonists approved for clinical use by the US FDA include cetrorelix (Cetrotide; Serono) and ganirelix (Antagon; Organon International). These agents are usually used as injectables. GnRH antagonists exert their action through the direct competitive inhibition of GnRH by occupying the pituitary GnRH receptors and therefore blocking the access of the endogenous GnRH and exogenously administered agonists to their receptor sites [38, 39]. These agents may induce a deep suppression of gonadotropins and the sex steroids, while avoiding any “flare up” phenomena, which may lead to a reduction in uterine fibroids size of up to 50% [40]. One of the major limitations to the wide use of the GnRH antagonists in leiomyoma treatment is the short half-life of these agents and the non-availability of the Depot formulation, thus require repetitive dosing (daily for most of the antagonists) [41]. 

### 5.1. Promising GnRH Antagonist (Elagolix)

Elagolix is a second-generation new nonpeptide (GnRH) antagonist, highly potent antagonist orally active and rapidly bioavailable after administration that is being developed by Abbott Laboratories (Abbott) in collaboration with Neurocrine Biosciences [42, 43]. It is finalizing the Phase III for endometriosis and finalizing Phase II for uterine leiomyoma with opportunity to be its first and only approved oral treatment for uterine leiomyoma [44]. This promising compound inhibits gonadotropin releasing hormone (GnRH) receptors in the pituitary gland leading to a dose-dependent suppression of LH, FSH, and estradiol. Consequently, suppression of E2 is more prolonged at higher doses [45]. Pituitary suppression is maintained for only a portion of the day, and baseline gonadotropin levels return by 24 hours [46].

These properties suggest that Elagolix may enable dose-related pituitary and gonadal suppression in premenopausal women as part of treatment strategies for reproductive hormone-dependent disease states [46]. To date, Elagolix has been studied in 18 clinical trials totaling more than 1,000 subjects.

Elagolix seems to be well tolerated for multipledoses up to 200; rapidly absorbed after oral administration, with median time of maximum plasma concentration (Tmax) values ranging from 0.5 to 1 h, the primary metabolite (NBI-61962) appears in the serum rapidly after administration [46].

The therapeutic window of E2 levels for suppression of endometriosis is attainable at a dose of 100–150 mg/day with serum estradiol remained between 20 and 50 pg/mL [45]. This is supported by Barberi RL findings which showed that E2 levels between 30 and 50 pg/mL are effective in inducing endometrial atrophy [47]. The Elagolix therapeutic dose for management of uterine fibroid is yet to be determined.

## 6. Selective Estrogen Receptor Modulator (SERMs)

Selective estrogen receptor modulators (SERMs) are nonsteroidal estrogen receptor ligands that display tissue-specific agonist-antagonist estrogenic actions. They are used frequently in the treatment and prevention of estrogen receptor-positive carcinoma of the breast in addition to their use as ovulation induction agents [48, 49]. Tamoxifen is one of the oldest known SERMs, but it may potentially cause endometrial carcinoma due to its partial agonistic effect on the endometrium [50]. There are no randomized controlled trials that have investigated the potential role of Tamoxifen in the treatment of uterine fibroids; however, a few case reports have suggested that it actually increases leiomyoma growth [48, 51]. Raloxifene is another SERM that can be theoretically considered to be a candidate therapeutic option for uterine fibroids. Raloxifene only slightly affected collagen biosynthesis in control myometrium cells; however, it significantly inhibited collagen biosynthesis in leiomyoma cells [52] and exerted its action at the transcriptional level [53]. A newly developed SERM, “Lasofoxifene”, is currently awaiting FDA approval. However, the results of early trials suggest that there were no significant benefits compared to raloxifene for the skeleton, breast, heart, or reproductive tract [54, 55].

### 6.1. Mechanism of Action

The most probable hypothesis that explains SERMs' mechanism of action is that they induce changes in estrogen receptors, which result in differential expression of specific estrogen-regulated genes in different tissues [56]. Every member of the SERM family has its own individual characteristics, which depend on its structure, the type of estrogen receptor they bind to, and the set of molecules that interact with its estrogen receptor/SERM complex in affected cells, and these characteristics result in either agonistic or antagonistic activity [57]. SERMs could potentially provide therapeutic benefits by having antagonistic effects at uterine myometrial level and by preventing ovarian stimulation which has been achieved in rat studies. The difference in activity of SERMs is based on the structure activity relationships (SARs) [58].

### 6.2. SERMs and Treatment of Uterine Fibroids

All SERMs, with their estrogen blocking activity, would be theoretically expected to exert at least some therapeutic effect on uterine fibroids. Raloxifene has been shown to enhance the shrinkage of uterine fibroids in postmenopausal women [59, 60]. However, a recent report from Italy that addressed the effect of raloxifene on uterine leiomyoma showed that the leiomyoma size in premenopausal women who were administered daily 60 mg doses of raloxifene over a 2-year period exhibited no change in leiomyoma size [61].

### 6.3. Adverse Events

Tamoxifen is not recommended for women with a prior history of deep venous thrombosis, pulmonary embolus, stroke, or transient ischemic attack because it increases the risk of ischemic stroke, particularly in women who are 50 years of age or older. Additionally, the risk of uterine/endometrial cancer was approximately doubled with tamoxifen use [62], and the risk of superficial thrombophlebitis was three times higher [41, 50]. Some of these side effects could be explained by the inhibition of cellular glutamine uptake, oxidative stress, and the induction of apoptosis [63]. SERMs are seldom used for the treatment of uterine fibroids [52].

## 7. Aromatase Inhibitors

Aromatase inhibitors (AIs) significantly block both ovarian and peripheral estrogen production within 1 day of treatment [64]. Letrozole suppressed the production of estrogens, particularly estrone and estradiol, by 76–79% compared to their baseline levels [65]. The underlying mechanism is the inhibition of the aromatase enzyme, which is the enzyme that catalyzes the conversion of androgenic substances into estrogens [66]. Recent reports have suggested that aromatase is expressed to a greater extent in uterine leiomyoma tissues of African-American women compared to Caucasian women, which may contribute to the higher incidence of ULMs in African American women [67]. Aromatase inhibitors have been shown to be effective against fibroids in limited short term studies with dosing regimens that included 2.5 mg per day of letrozole and 1 mg per day of anastrozole [68]. One of the major concerns with the use of aromatase inhibitors is the reported bone loss with prolonged use, which necessitates the concomitant use of oral contraceptive pills or progesterone [69]. A recently published RCT compared the effects of three months of aromatase inhibitor (letrozole) to that of three months of gonadotropin-releasing hormone agonist (triptorelin) on uterine leiomyoma volume and hormonal status [70]. The results showed an advantage of the rapid onset of action of AIs in addition to the avoidance of the flare ups that initially occurs with GnRHa. Both treatment options induced significant shrinkage of the uterine fibroids and improvement in leiomyoma-associated symptoms [70]. The mean reduction of leiomyoma volume with 3-month use of anastrozole is 55.7% [71]. The authors suggested that aromatase inhibitors should be considered in women with fibroids on a short-term basis or in women who want to avoid surgical intervention to preserve their potential fertility [72]. Another concern with the use of AIs as a treatment option for uterine leiomyoma is its off-label use, which mandates a thorough review with patients prior to the initiation of the therapy [69]. Several RCTs are underway that would hopefully add to our understanding of the potential promising role of AIs in the treatment of uterine leiomyomas [62].

## 8. Antiprogesterones

Estrogen has traditionally been considered to be the most important stimulus for leiomyoma growth and numerous studies that included cell culture and animal models supported this concept [73]. Surprisingly, recent findings suggest that volume maintenance and growth of human ULMs are also heavily progesterone dependent, and hence antiprogesterone could reverse leiomyoma growth effects [74, 75]. One potential link between the effects of the two key steroid hormones on ULMs is that estradiol induced the expression of the progesterone receptor and supported progesterone action on leiomyoma tissue [73]. Clinical findings also support these laboratory observations; studies have involved the evaluation of mifepristone (RU 486) [76–78], azoprisnil [68, 75], and, more recently, CDB-2914 and CDB-4124 (CDB: Contraceptive Development Branch) [79].

### 8.1. Mifepristone

Mifepristone (RU486), a well-known oral antiprogesterone compound, has been used for more than 20 years for multiple clinical indications [70, 80–82]. It has recently been evaluated as a potential therapeutic agent for uterine fibroids with a dose that ranges from 5 mg to 50 mg over a 3-month period [83–85]. Mifepristone reduced leiomyoma size (26% to 74%) and improved leiomyoma-related symptoms (63% to 100% induction of amenorrhea). Reported side effects included transient elevations in transaminases, which occurred in 4% of cases as well as endometrial hyperplasia and was detected in 28% of the women who were screened with endometrial biopsies [86]. However, these studies were mostly preliminary with limited numbers of subjects, and therefore, larger randomized well-controlled trials that include thorough monitoring of liver function and endometrial histology are required to conclusively determine the safety and efficacy of this treatment modality.

### 8.2. Asoprisnil

Asoprisnil (J867, BAY86-5294) is an investigational selective progesterone receptor modulator (SPRM) that was developed for the treatment of progesterone-sensitive myomata. It induces unique morphological changes and is associated with inhibited proliferation of the endometrium and leiomyomata. These changes may lead to amenorrhea, which is usually encountered with its use [68, 87, 88]. Asoprisnil is a tissue selective molecule that binds to the progesterone receptors with a threefold greater affinity than endogenous progesterone [83]. It reduces the uterine and leiomyoma volumes in a dose-dependent manner while achieving remarkable decreases in menorrhagia scores in women with menorrhagia [89]. Amenorrhea rates also increased as the dose of asoprisnil was increased [84, 87]. When asoprisnil was administered daily for longer than 3-4 months, significant endometrial thickening and unusual histological appearance of the endometrial glands occurred [85].

### 8.3. Telapristone Acetate/CDB-4124 (Proposed Trade Names, Proellex, Progenta)

CDB-4124 is another SPRM, but it is a relatively pure progesterone antagonist. It was studied in recent years for the treatment of uterine fibroids and is still being evaluated to address its safety and dose parameters in premenopausal women [90]. Limited information or publications are currently available on the various clinical trials that have investigated CDB-4124; these studies have either been completed or were terminated due to adverse liver-related events according to the http://www.clinicaltrials.gov/ website. New clinical trials using lower doses of CDB-4124 have recently been approved by the FDA.

### 8.4. Ulipristal/CDB-2914 (VA 2914, EllaOne, Ella)

Ulipristal is an FDA-approved selective progesterone receptor modulator (SPRM) that is indicated for emergency contraception. It is structurally similar to mifepristone and seems to be effective in the treatment of uterine fibroids. It is associated with a reduction in pain, bleeding, and leiomyoma size between 17 and 24% [91], as well as an improvement in quality of life [92]. However, data on long-term treatment are lacking, and similar to other SPRMs, ulipristal may be associated with endometrial thickening and endometrial hyperplasia [85, 93, 94]. Large randomized well-controlled clinical trials are needed to evaluate the utility of ulipristal for potential clinical treatment of uterine fibroids [93].

## 9. Somatostatin Analogues

Increasing evidence has demonstrated a role for growth factors, such as insulin growth factor I (IGF-I) and IGF-II, in the initiation and progression of uterine fibroids [95–98]. Leiomyoma tissue expresses higher levels of IGF-I/IGF-II receptors compared to normal adjacent myometrium [89, 97]. Additionally, these tissues secrete their own IGF-1, probably for autocrine and paracrine use [98]. From a clinical perspective, it has been recently reported that patients with high levels of growth hormone (acromegalic patients) have a higher prevalence of uterine fibroids than the general population [99]. Lanreotide, which is a long-acting somatostatin analogue that has been shown to reduce growth hormone secretion, has also recently been evaluated in seven women with uterine fibroids in Italy [100]. Interestingly, lanreotide induced a 42% mean myoma volume reduction within a 3-month period. These results show that somatostatin analogues may potentially be a new therapy for uterine fibroids [101]. The treatment with somatostatin analogues for diseases other than leiomyoma appears to be safe and is usually well tolerated with some reports of gallstone formation [102, 103]. However, the lacking of clinical trials which test the long-term use of somatostatin analogues along with the severe and adverse health implications such as decreased life expectancy due to accelerated heart disease observed in adults with growth hormone deficiency may hinder its future use for leiomyoma treatment.

## 10. Cabergoline

Carbergoline is a well-known dopamine agonist that is effectively used in the treatment of prolactinoma and for the inhibition of lactation. A recent study [104] evaluated carbergoline as a therapeutic option for uterine fibroids. The rational for such an approach lies in its effect as an inhibitory agent on GnRH release. A group in Iran published a preliminary study in 2007 [104] that favored the use of carbergoline as a medical treatment of uterine fibroids on which they reported a volume reduction of about 50% with 6-week use [92]. The same group performed a subsequent study that compared carbergoline with diphereline, which is a gonadotropin-releasing hormone agonist [105]. They reported comparable results in terms of the shrinkage of the fibroids and the improvement in the sonographic, clinical, and intraoperative outcomes [105]. These findings warrant future larger controlled trials to clearly assess the potential use of carbergoline in the treatment of uterine fibroids.

## 11. Danazol

Danazol is a synthetic steroid that inhibits steroidogenesis through multienzymatic actions in addition to its suppressor effect on sex hormone binding globulin [106]. It reportedly induced a significant 24% volume reduction [107, 108]. However, a recent Cochrane study failed to identify any randomized controlled trials that compared danazol to placebo or any other medical therapy in women with uterine fibroids [109].

## 12. Gestrinone

Gestrinone is a steroid that possesses antiestrogen receptor and antiprogesterone receptor properties in various tissues, including the endometrium [110]. A recent report from Italy evaluated the use of Gestrinone in the treatment of premenopausal women with uterine fibroids at a dose of 2.5 mg twice per week over a 6-month period [110]. The authors reported a 32% ± 10% reduction in uterine volume [110]. A subsequent study reported up to 60% leiomyoma shrinkage in size [111]. Gestrinone is a contraceptive agent and also exhibits several unfavorable side effects, such as mild androgenicity, weight gain, seborrhea, acne, hirsutism, and occasional hoarseness.

##  Disclosure

Dr. A. Al-Hendy was a site principal investigator in phase III clinical trials of “Azoprisnil” and “Pro-ellex”. Dr. Mohamed Sabry has nothing to disclose.

## Figures and Tables

**Table 1:** The clinical presentation of uterine leiomyomas.

(i) Asymptomatic
(ii) Abnormal uterine bleeding
(a) Menorrhagia
(b) Anemia
(iii) Pelvic pressure
(a) Urinary frequency
(b) Urinary incontinence
(c) Difficulty with urination
(d) Hydronephrosis
(e) Constipation
(f) Tenesmus
(iv) Pelvic mass
(v) Pelvic pain
(vi) Infertility
(vii) Obstetric complications
(viii) Pregnancy related
(a) Myoma growth
(b) Red degeneration and pain
(c) Spontaneous miscarriage
(ix) Malignancy
(x) Rare associations
(a) Ascites
(b) Polycythemia
(c) Familial syndromes, renal cell carcinoma
(xi) Benign metastasizing

**Table 2:** Diagnosis of uterine leiomyoma.

(i) Pelvic examination: enlarged, irregular, firm, nontender uterus
(ii) Ultrasound: transvaginal ultrasound, hypoechoic, heterogenous masses
(iii) Saline sonohysterography: for submucous fibroids or polypi
(iv) MRI: best method for exact mapping, numbering of fibroids
(v) Hysteroscopy: diagnosis of submucous fibroids
